# Quercetin as a potential treatment for COVID-19-induced acute kidney injury: Based on network pharmacology and molecular docking study

**DOI:** 10.1371/journal.pone.0245209

**Published:** 2021-01-14

**Authors:** Yue-Yu Gu, Min Zhang, Huan Cen, Yi-Fan Wu, Zhaoyu Lu, Fuhua Lu, Xu-Sheng Liu, Hui-Yao Lan

**Affiliations:** 1 Department of Nephrology, The Second Affiliated Hospital of Guangzhou University of Chinese Medicine, Guangdong Provincial Hospital of Chinese Medicine, Guangzhou, China; 2 Department of Ultrasound, The Second Affiliated Hospital of Guangzhou University of Chinese Medicine, Guangdong Provincial Hospital of Chinese Medicine, Guangzhou, China; 3 Department of Medicine and Therapeutics, Li Ka Shing Institute of Health Sciences, The Chinese University of Hong Kong, Hong Kong, China; University of Florida, UNITED STATES

## Abstract

Kidneys are one of the targets for SARS-CoV-2, it is reported that up to 36% of patients with SARS-CoV-2 infection would develop into acute kidney injury (AKI). AKI is associated with high mortality in the clinical setting and contributes to the transition of AKI to chronic kidney disease (CKD). Up to date, the underlying mechanisms are obscure and there is no effective and specific treatment for COVID-19-induced AKI. In the present study, we investigated the mechanisms and interactions between Quercetin and SARS-CoV-2 targets proteins by using network pharmacology and molecular docking. The renal protective effects of Quercetin on COVID-19-induced AKI may be associated with the blockade of the activation of inflammatory, cell apoptosis-related signaling pathways. Quercetin may also serve as SARS-CoV-2 inhibitor by binding with the active sites of SARS-CoV-2 main protease 3CL and ACE2, therefore suppressing the functions of the proteins to cut the viral life cycle. In conclusion, Quercetin may be a novel therapeutic agent for COVID-19-induced AKI. Inhibition of inflammatory, cell apoptosis-related signaling pathways may be the critical mechanisms by which Quercetin protects kidney from SARS-CoV-2 injury.

## Introduction

The international outbreak of coronavirus infection disease 2019 (COVID-19) is caused by the severe acute respiratory syndrome coronavirus 2 (SARS-CoV-2) [1, 2], which leads to the severe infection in the respiratory system and induces comorbidities with multiple organ dysfunction [3]. As of 15^th^ December 2020, COVID-19 had been tracked 1.62 million deaths and 72.8 million confirmed cases worldwide. Emerging evidence had shown that kidneys are the one of the targets for SARS-CoV-2, suggesting the acute kidney injury (AKI) as the fatal outcomes of COVID-19 [4]. Besides, hospitalized patients with COVID-19 had shown a high prevalence of developing into AKI, approximately 36.6% of the confirmed patients developed into AKI [5, 6]. Nevertheless, patients who heal from the disease are still in a high risk of developing into CKD due to the irreversible damage to kidneys caused by SARS-CoV-2 infection [7].

Although the underlying mechanisms linking COVID-19 and the incidence of AKI are still poorly understood, the significant roles of renal tubular, podocytes, other resident cells and inflammatory cells in mediating this link is gaining support by latest studies [8, 9]. Clearly, the direct infection of the kidneys by SARS-CoV-2 had been confirmed by using light and electron microscopy, that the presence of viral particles was observed in the renal tubular epithelium [10]. Of note, direct evidence also had shown the invasion of coronavirus particles in podocytes, as well as the positive staining of SARS-CoV nucleoprotein antibody in renal tubules [10, 11]. Moreover, as one of the main SARS-CoV-2 receptors, the angiotensin-converting enzyme 2 (ACE2) is highly expressed in the kidneys [12]. Recent evidence had also proven an upregulated expression of ACE2 in the proximal tubular cells [11]. As important receptor protein for coronavirus, SARS-CoV-2 main protease 3CL (3CLpro) plays a vital role in the life cycle of SARS-CoV-2 for endocytosis [13]. These two receptors are of great significance to provide drug targets to halt the progression of COVID-19.

Nevertheless, renal damages in COVID-19 patients may also be induced by renal-toxic antiviral compounds, complement activation, aggressive inflammation and deficiency in blood oxygen supply [14, 15]. With the elevating baseline serum creatinine (SCr), blood urea nitrogen (BUN), proteinuria, and hematuria, patients with renal disease were in higher risk for the in-hospital death [16]. Until now, the pandemic COVID-19 continue to spread around the world with substantial morbidity and mortality, however, no specific vaccine or safe and effective therapies are available for the treatment against the coronavirus infection. Therefore, we are in urgent need of medications and treatment options for COVID-19 and comorbidities.

It is reported that herbal medicines play a protective role in the treatment of patients infected with SARS-CoV-2, highlighting the possibility of herbal compounds as one of the promising drugs for COVID-19 and its comorbidities [17]. Quercetin is a naturally abundant flavonoid that widely distributes in various herbal medicines, which had been predicted as one of the potential antiviral drugs that might halt the coronavirus infection via multiple signaling pathways [18]. Based on the supercomputer SUMMIT drug-docking screen and expression profiling experiments of Gene Set Enrichment Analyses (GSEA), Quercetin was listed as one of the promising compounds to serve as scaffolds that could inhibit the infection of SARS-CoV-2 [19]. The protective role of Quercetin in kidney diseases had been reported by a number of studies [20–23]. Notably, Quercetin was demonstrated as effective drug on ameliorating AKI by modulating the polarization of M1/M2 macrophage and Mincle/Syk/NF-κB signaling-mediated macrophage inflammation [24, 25]. These findings have proven Quercetin as reasonable drug for COVID-19-induced AKI.

**Fig 1:**
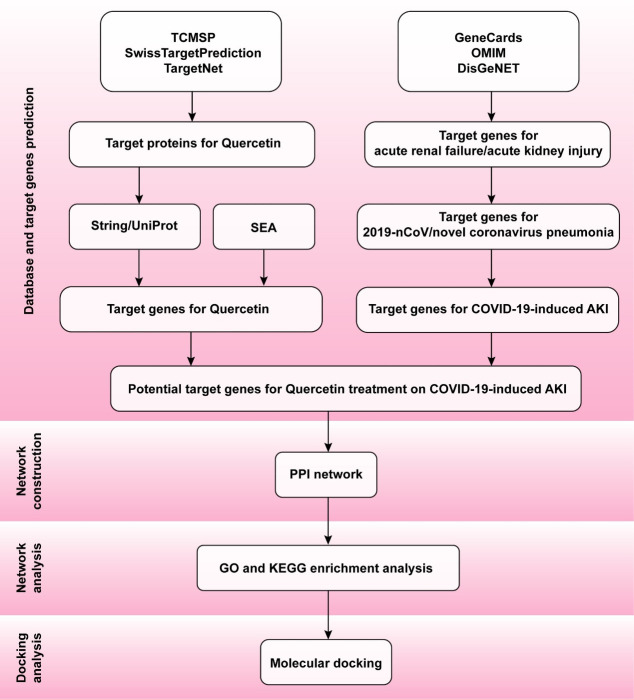
General workflow of network pharmacology and molecular docking in the present study.

In our study, the potential effects and mechanisms of Quercetin on COVID-19-induced AKI were analyzed by network pharmacology. Molecular interactions between Quercetin and SARS-CoV-2 target receptors were studied by molecular docking (Fig 1).

## Materials and methods

### Prediction of Quercetin-associated target genes and their intersection on COVID-19-induced acute kidney injury

The absorption, distribution, metabolism, excretion (ADME), oral bioavailability (OB), and drug-likeness (DL) information of Quercetin was obtained on TCMSP database (http://tcmspw.com/tcmsp.php) [26]. Quercetin is a polyphenolic flavonoid with a molecular weight of 302.23g/mol, pharmacological properties, chemical structure and molecular formula of Quercetin were indicated in S1 Table and S1 Fig. Target proteins of Quercetin were obtained in the TCMSP database. The SMILES structural formula of Quercetin was downloaded from PubChem database (https://www.ncbi.nlm.nih.gov/pccompound) [27]. The SMILES structural formula was input in the SwissTargetPrediction database (http://swisstargetprediction.ch/) and TargetNet database (http://targetnet.scbdd.com/home/index/) to obtain the target proteins with a probability> 0 [28], and the SEA database (http://sea.bkslab.org) to obtain the corresponding target genes. The corresponding target genes were obtained associated with their target proteins through the String database (https://string-db.org/) [29] and UniProt databases (https://www.uniprot.org/) [30].

Target genes related to new coronary pneumonia were obtained from GeneCards (https://www.genecards.org/) [31], OMIM (https://omim.org/) [32], and DisGeNET (https://www.disgenet.org/) [33] databases by using keywords such as "2019-nCoV, novel coronavirus pneumonia or COVID-19". Target genes referred to acute renal failure and/or acute kidney injury were obtained. Next, the potential target genes of new coronary pneumonia, acute renal failure, and acute kidney injury were combined and recorded into documents of txt. format. The Venn analysis tool [34] was utilized to obtain the combination target genes of acute renal failure, acute kidney injury, and COVID-19, this cluster of targets gene was regarded to be the kidney damage-related genes which were induced by COVID-19. The cluster was further combined with the target genes of Quercetin to get a new cluster, which is referred to the cluster of Quercetin-related potential target genes on COVID-19-induced AKI. A Venn diagram was plotted using the OmicShare platform, a free online platform for data analysis (http://www.omicshare.com/tools).

### Construction of the Protein-Protein Interaction (PPI) network and analysis on gene ontology (GO) enrichment and KEGG pathways

The cluster of target genes of Quercetin, COVID-19, ARF (acute renal failure), and AKI were imported to the String database, "homo sapiens" was defined as current setting, and the confidence level was set to 0.4 to obtain the relationship on protein interactions. The result was saved as a file of TSV format and imported into Cytoscape 3.6.0 to build a PPI network [35].

The cluster of target genes of Quercetin, COVID-19, ARF, and AKI were analyzed by DAVID database and the species was set to "homo sapiens" [36]. The gene ontology (GO) enrichment analysis and Kyoto encyclopedia of genes and genomes (KEGG) pathway analysis were performed. The GO enrichment analysis includes biological process (BP), cell component (CC), and molecular function (MF). Top 15 GO analysis results together with their significant P value were selected to draw a histogram; the results of top 20 KEGG pathways with significant P value were also obtained to draw a bubble chart. A *P* value ≤0.05 was considered significant. A bubble chart was plotted using the OmicShare platform, a free online platform for data analysis (http://www.omicshare.com/tools).

### Molecular docking

The underlying mechanisms and interactions between Quercetin, COVID-19-related target proteins such as SARS-CoV-2 main protease 3CL (PDBID: 6LU7) and ACE2 (PDBID: 1R42) may be revealed and predicted by docking strategy. Molecular docking was applied to verify and test how Quercetin (ligand) interacts with target proteins (receptors). The 3D crystal structure of Quercetin (CID: 5280343) was obtained from the PubChem (http://pubchem.ncbi.nlm.nih.gov/compound/). 3D structure of Quercetin has been shown in **Fig 6A**. The 3D crystal structure of receptors SARS-CoV-2 main protease 3CL and ACE2 were selected from Protein Data Bank (PDB) (http://www.rcsb.org/pdb/), as shown in **Figs** 6**B** **and** 7A, respectively.

Ligand and proteins were prepared and docked by the Autodock 1.5.4 tools (Molecular Graphics Laboratory, the Scripps Research Institute). AutoDockTools 1.5.6. The results were shown with binding energy (BE), a weighted average of docking score, to assess the reliability and describe the accuracy of the ligand positioning. The more negative energy is, the better the ligand [37]. The BE of Quercetin and two target proteins were shown in S1 Table. The docking results were analyzed in Discovery Studio (DS) 2.5 (Accelrys Software Inc., San Diego, U.S.A.) to evaluate the potential interactions of ligand and the proteins. The DS program was run by using a local host 9943 server on the system of Microsoft Window 7 according to previous protocol [38].

The datasets downloaded from the databases in the present study have been uploaded and can be accessed by this link: https://doi.org/10.6084/m9.figshare.13379273.v1

## Results

### Potential Quercetin-related target genes in COVID-19-induced AKI

Quercetin appears as yellow needles or yellow powder. It converts to anhydrous form at 203–207°F. The pharmacokinetic properties of Quercetin were shown in S1 Table. The OB and DL are important information in pharmacology. On one hand, OB refers to the relative amount of drug absorbed into the systemic blood circulation after administration by extravascular route. One the other hand, DL refers to the similarity of a compound to a known drug. The standard criteria were described as OB≥30% and DL≥0.18 [39, 40]. In our study, the OB and DL of Quercetin is 46.43% and 0.28%, respectively.

**Fig 2:**
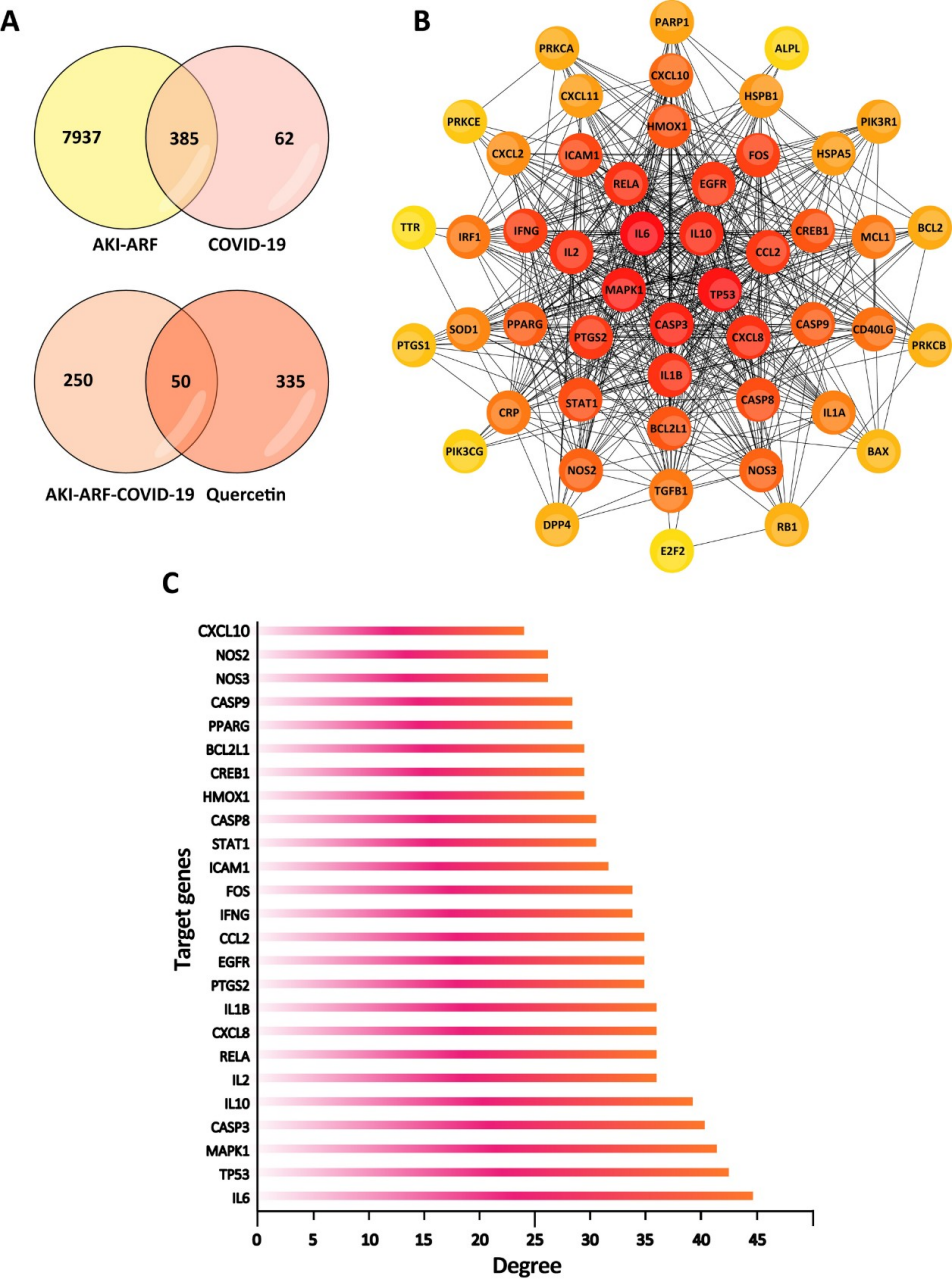
Potential Quercetin-related target genes in COVID-19-induced AKI. (A) Venn diagram of the intersection relationship of target genes between Quercetin, AKI-ARF and COVID-19. (B) PPI network of potential target genes related to Quercetin treatment on COVID-19-induced AKI. (C) 25 significant target genes with a degree score over 22.52 points were indicated to take part in the process of COVID-19-induced AKI with Quercetin treatment.

A total of 317 target proteins were collected from TCMSP, SwissTargetPrediction and TargetNet databases. We obtained 300 significant target genes from String, Uniprot, SEA databases. From the GeneCards, OMIM, and DisGeNET databases, 447 COVID-19-related target genes and 8323 target genes responsible for ARF and AKI were collected. 385 target genes were joint genes in the two clusters of AKI-ARF and COVID-19-related genes. Furthermore, among these 385 potential target genes, 50 out of them were analyzed to be significantly responsible to Quercetin treatment on COVID-19-induced AKI (Fig 2A), detail information on these target genes were shown in Table 1.

**Table 1:** Potential target genes of Quercetin treatment on COVID-19-induced acute kidney injury or acute renal failure.

No.	UniProtID	Gene Symbol	Gene name
**1**	P05231	IL6	Interleukin-6
**2**	P04637	TP53	Cellular tumor antigen p53
**3**	Q1HBJ4	MAPK1	Mitogen-activated protein kinase
**4**	P42574	CASP3	Caspase-3
**5**	P22301	IL10	Interleukin-10
**6**	P60568	IL2	Interleukin-2
**7**	Q04206	RELA	Transcription factor p65
**8**	P10145	CXCL8	Interleukin-8
**9**	P01584	IL1B	Interleukin-1 beta
**10**	P35354	PTGS2	Prostaglandin G/H synthase 2
**11**	E9PFD7	EGFR	Receptor protein-tyrosine kinase
**12**	P13500	CCL2	C-C motif chemokine 2
**13**	P01579	IFNG	Interferon gamma
**14**	P01100	FOS	Proto-oncogene c-Fos
**15**	P05362	ICAM1	Intercellular adhesion molecule 1
**16**	P42224	STAT1	Signal transducer and activator of transcription 1-alpha/beta
**17**	Q14790	CASP8	Caspase-8
**18**	Q96DI8	HMOX1	Heme oxygenase
**19**	P16220	CREB1	Cyclic AMP-responsive element-binding protein 1
**20**	Q07817	BCL2L1	Bcl-2-like protein 1
**21**	P37231	PPARG	Peroxisome proliferator-activated receptor gamma
**22**	P55211	CASP9	Caspase-9
**23**	P29474	NOS3	Nitric oxide synthase
**24**	P35228	NOS2	Nitric oxide synthase
**25**	P02778	CXCL10	C-X-C motif chemokine 10
**26**	P29965	CD40LG	CD40 ligand
**27**	P01137	TGFB1	Transforming growth factor beta-1
**28**	P02741	CRP	C-reactive protein
**29**	P10914	IRF1	Interferon regulatory factor 1
**30**	Q07820	MCL1	Induced myeloid leukemia cell differentiation protein Mcl-1
**31**	P01583	IL1A	Interleukin-1 alpha
**32**	P00441	SOD1	Superoxide dismutase [Cu-Zn]
**33**	P19875	CXCL2	C-X-C motif chemokine 2
**34**	P04792	HSPB1	Heat shock protein beta-1
**35**	P11021	HSPA5	Endoplasmic reticulum chaperone BiP
**36**	O14625	CXCL11	C-X-C motif chemokine 11
**37**	P09874	PARP1	Poly [ADP-ribose] polymerase 1
**38**	P27986	PIK3R1	Phosphatidylinositol 3-kinase regulatory subunit alpha
**39**	L7RSM7	PRKCA	Protein kinase C
**40**	P10415	BCL2	Apoptosis regulator Bcl-2
**41**	P27487	DPP4	Dipeptidyl peptidase 4
**42**	P05771	PRKCB	Protein kinase C beta type
**43**	P06400	RB1	Retinoblastoma-associated protein
**44**	Q07812	BAX	Apoptosis regulator BAX
**45**	P23219	PTGS1	Prostaglandin G/H synthase 1
**46**	Q02156	PRKCE	Protein kinase C epsilon type
**47**	P48736	PIK3CG	Phosphatidylinositol 4,5-bisphosphate 3-kinase catalytic subunit gamma isoform
**48**	P05186	ALPL	Alkaline phosphatase
**49**	P02766	TTR	Transthyretin
**50**	Q14209	E2F2	Transcription factor E2F2

### PPI networks

String database was used to obtain the protein and protein interactions of those 50 target genes. The result was then analyzed by Cytoscape 3.6.0 software and the PPI network was built (Fig 2B), Cytoscape plugin cytoHubba was used for ranking nodes in a network by their network features.

Degree is one of the 11 topological analysis methods provided by cytoHubba, target genes with higher degrees tend to be key target genes. Each node shows a different depth of color according to its own degree. The darker the color, the higher the degree [41]. The average degree was 22.52. There were 25 target genes with a higher degree than 22.52, and they were regarded as potential significant genes that play the key role in the mechanisms of Quercetin treatment to COVID-19-induced kidney injury (Fig 2C).

### GO enrichment analysis and KEGG pathway analysis

**Fig 3:**
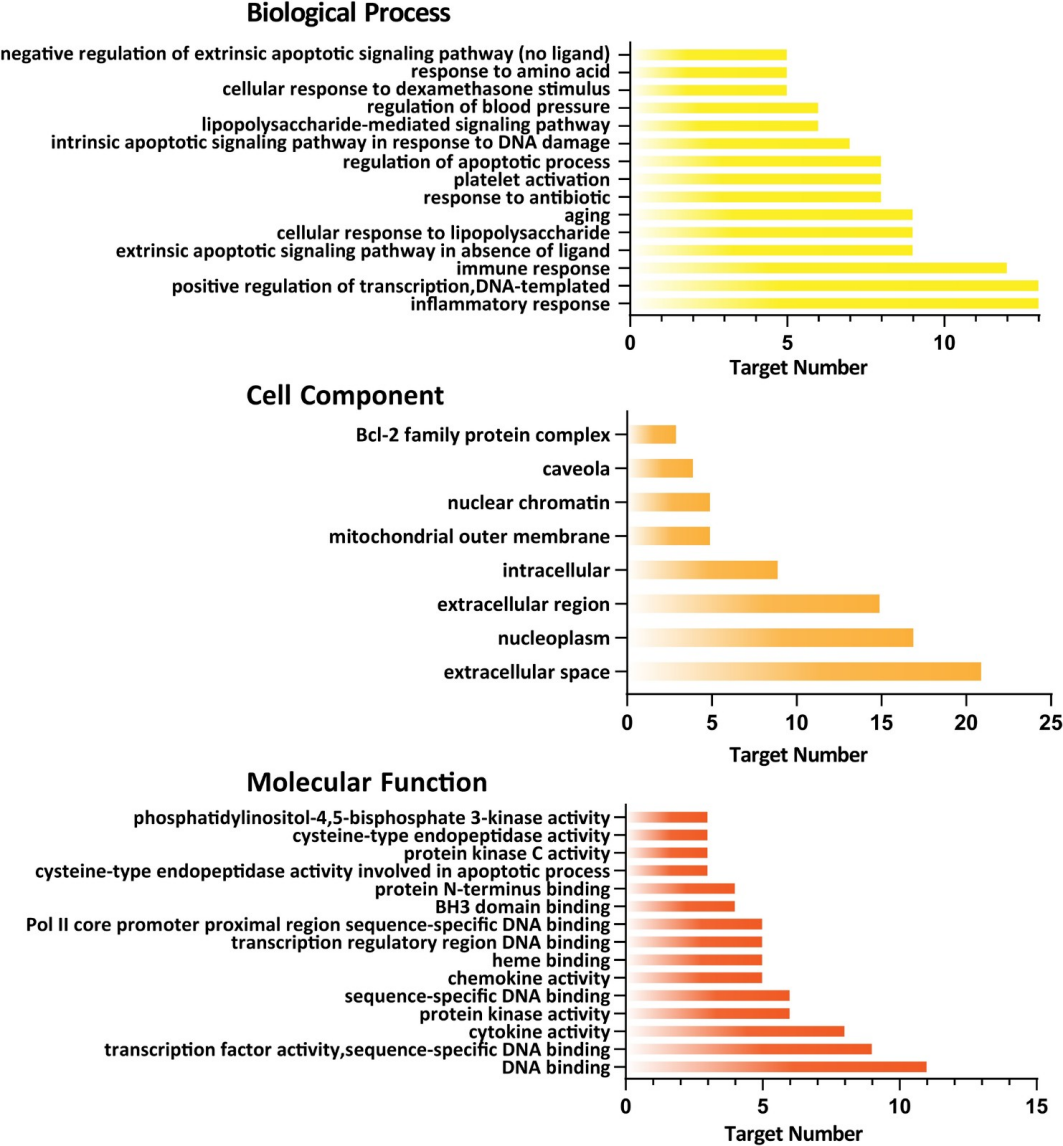
The gene ontology (GO) enrichment was performed on screened genes. The GO analysis had shown 59 entries on biological processes, 8 entries on cell components, and 19 entries on molecular functions with *P*<0.05. The top 15 entries with the most significant *P* value are shown.

**Fig 4:**
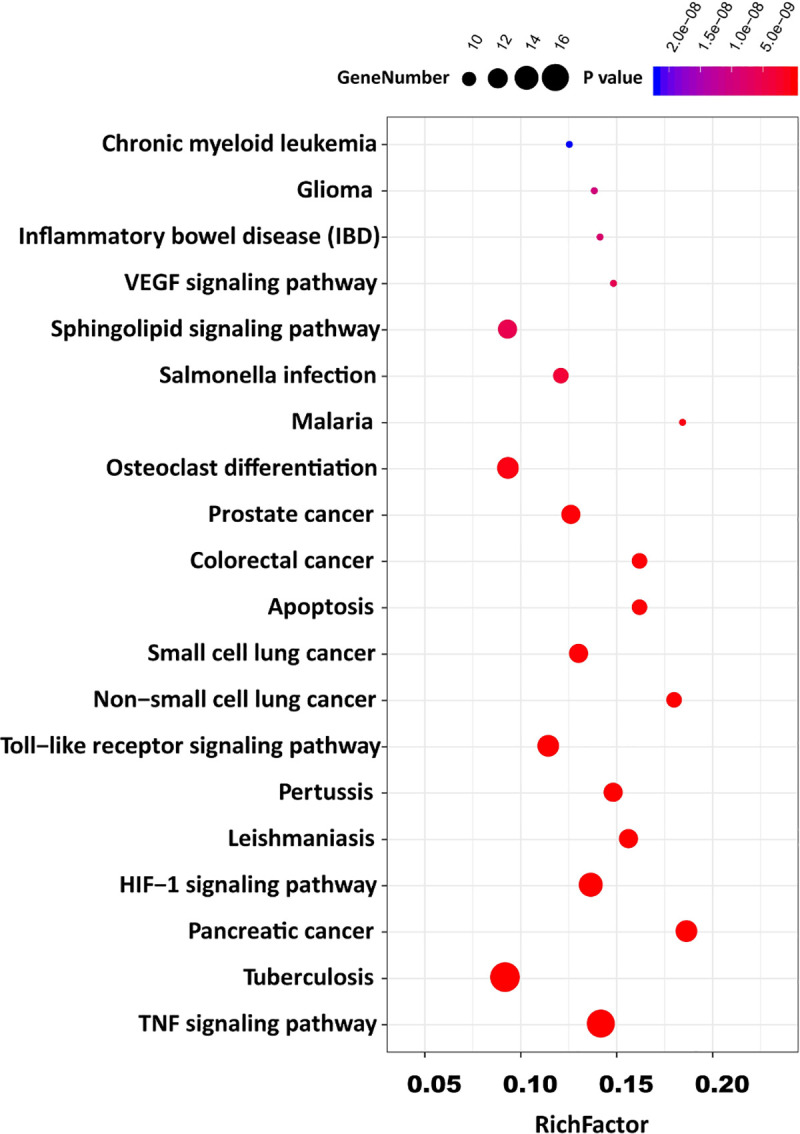
The top 20 potential KEGG pathway enrichment of screened target genes in COVID-19-induced acute kidney injury. Potential signaling pathways were shown as possible mechanisms and interactions in COVID-19-induced AKI. The index of rich factor represents the ratio of the number of the pathway-related target genes, and it represents the number of annotated genes in certain pathway, the higher score of rich factors, the higher level of enrichment. The size of the dots represents the number of target genes in their representative pathways, P values with scales were also highlighted with different colors as indicated on the top of the figure.

By GO analysis, a total of 86 GO items with P <0.05 were obtained, including 59 biological process entries, 8 cell component entries, and 19 molecular function entries. In biological processes, the target genes were involved in the regulation of apoptotic process, immune response, platelet activation, etc. In the cell components, the target genes potentially play roles in the extracellular space, extracellular region and nucleoplasm; In molecular functions, the target genes may play roles in the process of DNA binding, cytokine activity, protein kinase activity, etc. To better understand the gene ontology enrichment of these 50 target genes, top 15 entries with the most significant *P* value of each component were visualized and shown in Fig 3. A total of 84 KEGG pathways were also analyzed. The top 20 pathways with significant *P* value were converted into a bubble chart (shown in Fig 4).

### Potential targets and therapeutic pathways of Quercetin on COVID-19-induced acute kidney injury

**Fig 5:**
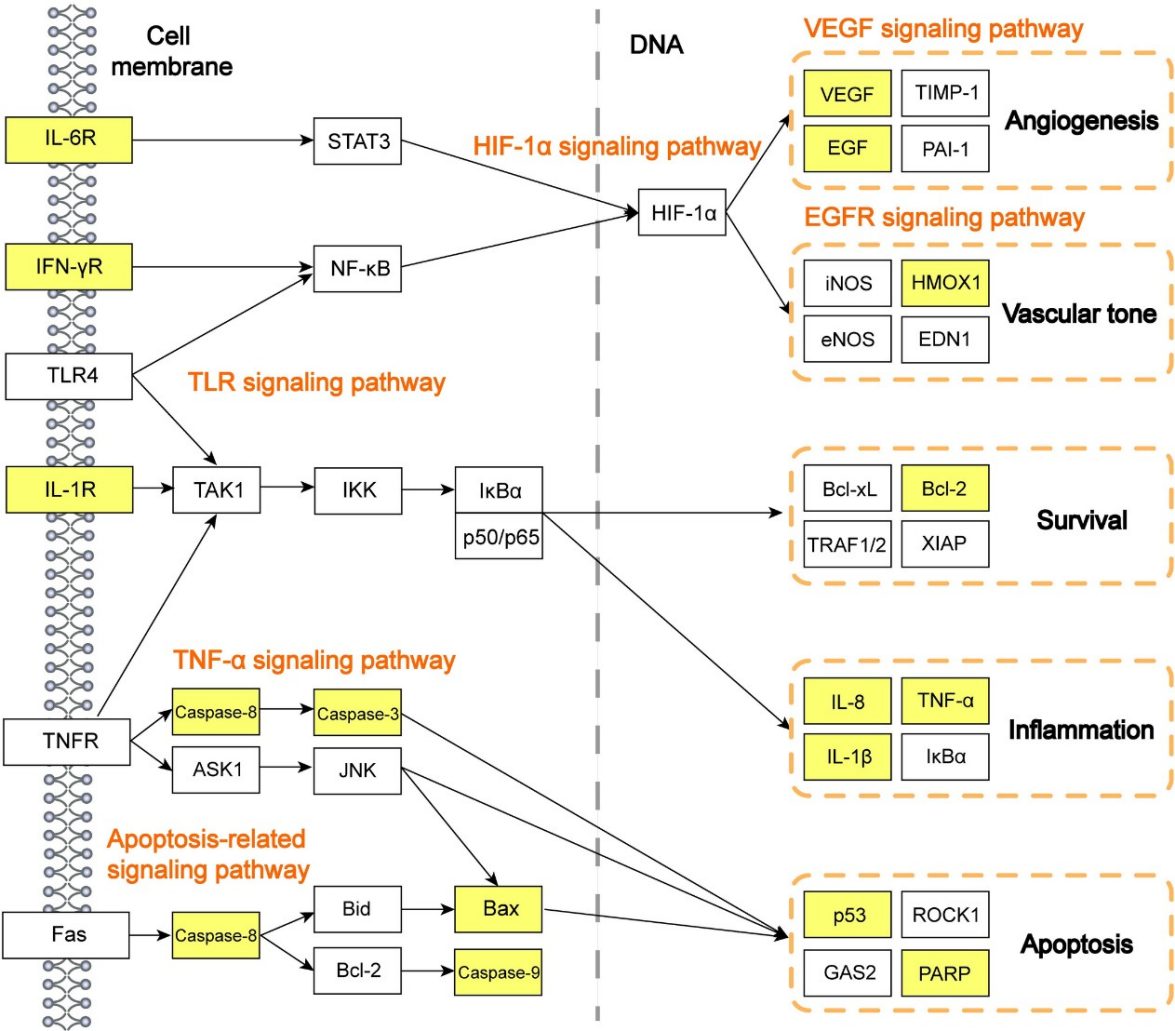
Systematic understanding of the potential targets and therapeutic pathways of Quercetin on COVID-19-induced AKI. All the indicated therapeutic pathways were concluded by published articles, nodes highlighted with yellow represent the Quercetin targets and other nodes represent the targets of COVID-19-induced acute kidney injury/acute renal failure.

Based on the current understanding of the pathogenesis of AKI or ARF, and the results of the KEGG pathway analysis, we further constructed the potential TNF, HIF-1α, Toll-like receptor (TLR), apoptosis-related, and VEGF signaling as the therapeutic pathways in Quercetin treatment to COVID-19-induced acute kidney injuries **(as shown in Fig 5)**. This network reveals significant potential signaling pathways involved in the pathogenic process of SARS-CoV-2 infected kidneys. Of note, it provides evidence on explaining that Quercetin may exert beneficial effects by improving angiogenesis, vascular tone, survival, inflammation, and apoptosis in AKI.

### Interactions and binding modes of Quercetin with COVID-19-related target proteins

**Fig 6:**
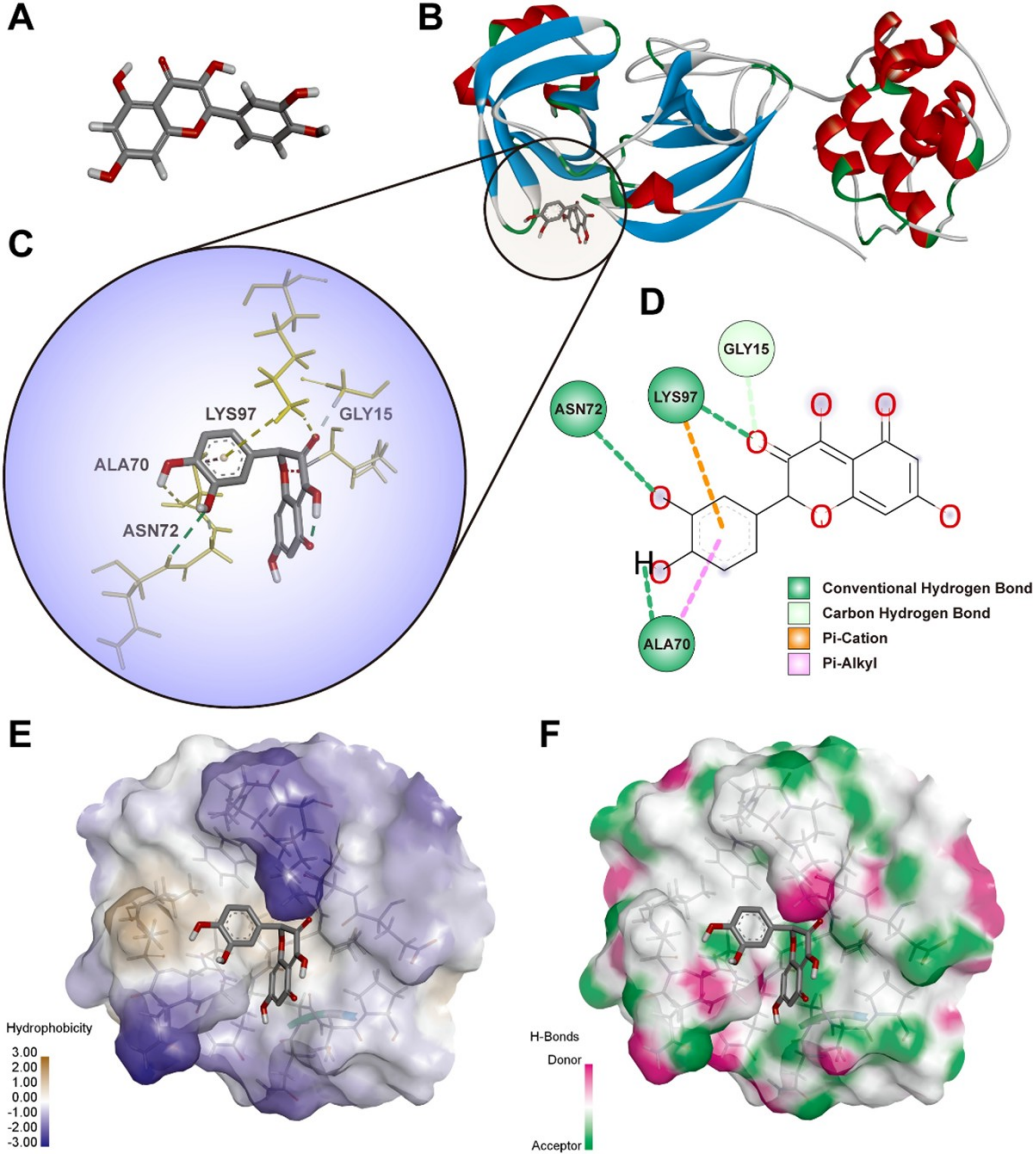
Diagrams of interaction of Quercetin with crystal structure of COVID-19 main protease 3CL (6LU7). (A) the 3D structure of Quercetin. (B) the human crystal structure of COVID-19 main protease 3CL (6LU7) with Quercetin as the ligand in the active binding site of 6LU7. (C) 3D docking pattern and molecular interactions 6LU7 with Quercetin. The interactive bonds are indicated by yellow dashed lines. (D) 2D docking pattern of Quercetin with amino acids ASN^72^, ALA^70^, LYS^97^ and GLY^15^ of 6LU7. (E) pocket view of Quercetin binding with 6LU7 and the hydrophobic surface. (F) pocket view of Quercetin binding with 6LU7 and the hydrogen bond donor-acceptor residues.

**Fig 7:**
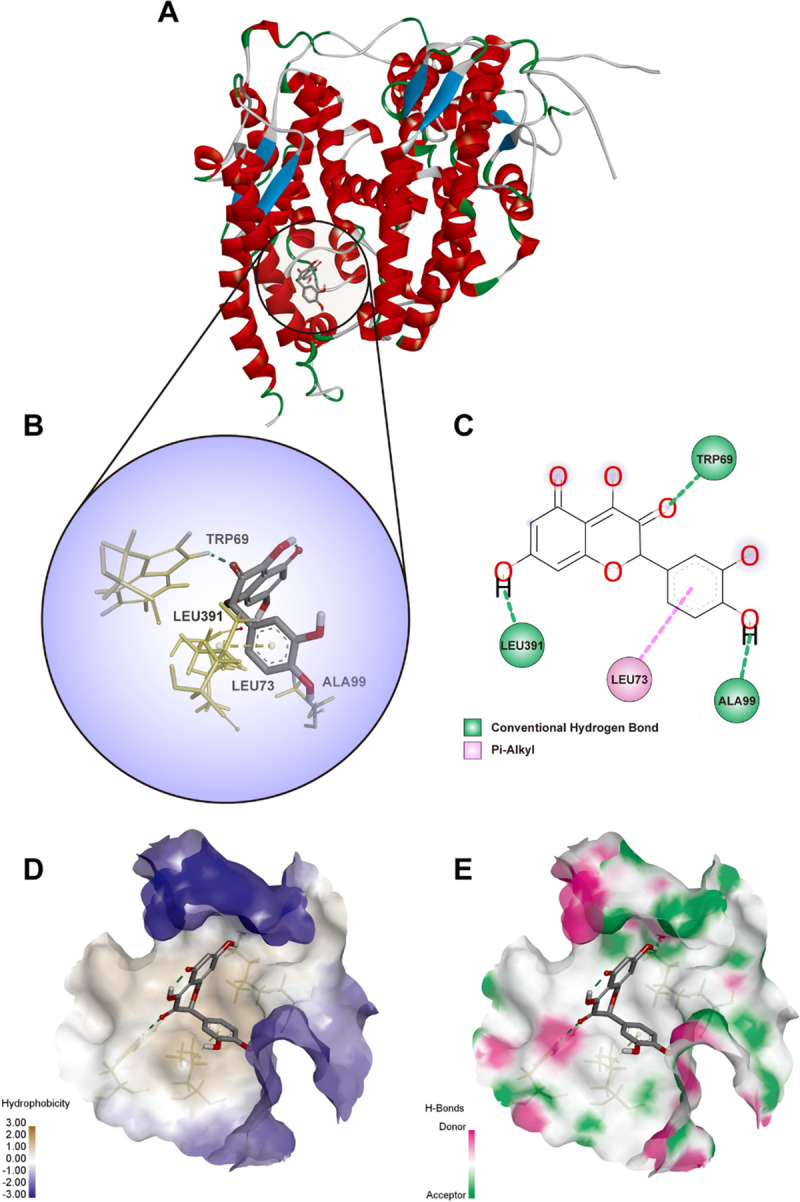
Diagrams of interaction of Quercetin with the crystal structure of ACE2 (1R42). (A) the human crystal structure of human ACE2 (1R42) with Quercetin as the ligand in the active binding site of 1R42. (B) 3D docking pattern and molecular interactions 1R42 with Quercetin. The interactive bonds are indicated by yellow dashed lines. (C) 2D docking pattern of Quercetin with amino acids TRP^69^, LEU^391^, LEU^73^ and ALA^99^ of 1R42. (D) pocket view of Quercetin binding with 1R42 and the hydrophobic surface. (E) pocket view of Quercetin binding with 1R42 and the hydrogen bond donor-acceptor residues.

By geometry and energy matching, the ligand and the receptor recognize each other and bind together. Molecular docking has provided a new insight into the ligand-receptor interactions and structural features of the two molecules within the active site [42, 43]. In our study, CDOCKER program was performed to analyze the interactions and binding features. As shown in **Figs 6B and 7A**, Quercetin **(3D structure shown in Fig 6A)** could be docked into the active sites of SARS-CoV-2 main protease 3CL (PDBID: 6LU7) and ACE2 (PDBID: 1R42) in the binding pocket, respectively. The 3D Quercetin was surrounded by amino acids of the target protein (6LU7) **(Fig 6C)**. Amino acid ASN^72^, ALA^70^, LYS^97^ and GLY^15^ formed the conventional hydrogen bond, carbon hydrogen bond, Pi-Cation and Pi-Alkyl interactions between Quercetin and SARS-CoV-2 main protease 3CL **(Fig 6D)**. The pocket views of the hydrophobic surface and hydrogen bond donor-acceptor residues had shown the binding of 6LU7 with Quercetin, as provided in **Fig 6E and 6F**. As for 1R42, Quercetin may be docked into the active site of receptor and form conventional hydrogen bond and Pi-Alkyl interactions by amino acids of TRP^69^, LEU^391^, LEU^73^ and ALA^99^ as shown in **Fig 7B and 7C**. The pocket view of hydrophobic and hydrophilic regions, hydrogen bond donor-acceptor residues between Quercetin and ACE2 (1R42) were shown in **Fig 7D and 7E**.

## Discussion

In the present study, we tried to find clues from the active compound—Quercetin, that it may serve as one of the promising drugs for COVID-19-induced AKI. By using *in silico* approaches such as network pharmacology and molecular docking, we elucidated the potential therapeutic network of COVID-19-induced AKI and the key signaling network of Quercetin treatment on this fatal disease. In addition, KEGG pathway analysis had indicated several signaling pathways, including TNF signaling, HIF-1α signaling, TLR signaling, VEGF signaling, apoptosis-related signaling pathways, and downstream molecules may play vital roles in the pharmacological mechanisms of Quercetin in treating COVID-19-induced renal injuries.

Based on current studies, Quercetin may be one of the key flavonoids that inhibits the coronavirus infective cycle with pleiotropic functions and low toxicity [44–46]. Luo L *et al*. had analyzed 179 single herbal medicines for treating COVID-19 in Chinese patients, the results have suggested Quercetin as the promising candidate for COVID-19 [47]. Besides, a number of latest network pharmacological studies also predicted Quercetin to be the most potential compounds in herbal formula or decoction that treated COVID-19 [48–51]. These findings have indicated that Quercetin could be an antiviral agent against SARS-CoV-2. Our results might support the previous findings on inflammation in virus-related acute injured kidneys. Increasing evidence shows that inflammatory and cytokine storm are associated with the severity of COVID-19 disease, for example, the level of Serum IL-6 and IL-10 are significantly higher in severe confirmed cases than in the mild group [52]. Previous studies have verified that Quercetin could play multiple regulatory roles in halting inflammation during AKI [24, 25]. All these findings have demonstrated the protective role of Quercetin in treating COVID-19-induced AKI.

SARS-CoV-2 main protease 3CL is the key player in the replication cycle of the virus, targeting 3CLpro is one of the therapeutic strategies to tackle the translation of viral RNA. Abian *et al*. identified Quercetin interacts with 3CLpro and affects the thermal stability of 3CLpro by using experimental and computational assay, providing evidence for Quercetin as potent inhibitor of SARS-CoV-2 main protease 3CL [53]. Other study also demonstrated that Quercetin might interact with amino acid residues GLU^288^, ASP^289^, GLU^290^ and ALA^285^ of the main protease. Interestingly, Quercetin also blocks the interaction sites of the viral spike protein [54]. In line with these findings, we studied and screened for the interactions between Quercetin and 3CLpro and found that Quercetin could be docked into the active site of 3CLpro and forming 4 types of bonds with amino acid ASN^72^, ALA^70^, LYS^97^ and GLY^15^. Investigation on the binding mode of SARS-CoV-2 main protease suggesting the potential clinical utility of Quercetin, and mutation on those interactive amino acids may also inhibit the activation and function of viral main protease.

ACE2 is the well-described entry receptor for SARS-CoV-2 in human cells, ACE2 is also expressed by vascular endothelial cells in kidneys. The binding interactions of spike glycoprotein and ACE2 receptors trigger a cascade of cytokine storm and inflammation, as well as the membrane fusion and internalization of the virus [55, 56]. Therefore, blocking the interaction of ACE2 with the S protein of SARS-CoV-2 could be effective therapeutics to inhibit viral infection and fatal inflammatory storm. In this study, we found that Quercetin is capable of binding with the active site of ACE2 by forming conventional hydrogen bond and Pi-Alkyl interactions with amino acids TRP^69^, LEU^391^, LEU^73^ and ALA^99^. Besides, the activation of TNF and NF-кB signaling pathways were identified as the novel changes in the pathogenesis of COVID-19 [57–59]. Our data from network pharmacology also suggested that five signaling pathways including TNF, HIF-1α, TLR, apoptosis-related, and VEGF signaling may be the therapeutic pathways in Quercetin treatment to COVID-19-induced AKI.

## Conclusion and future perspectives

The present study highlights the protective role of Quercetin in COVID-19-induced acute kidney injury by network pharmacology and molecular docking study, revealing the possible pathological mechanisms in renal injuries during coronavirus disease. Although the regulatory and mechanistic roles of Quercetin in COVID-19-induced AKI remains to be fully clarified, our study provides functional clues to suggest an alternative possibility in developing Quercetin into the promising therapeutic agent to combat the current pandemic. However, there are several limitations in the current study, including the lack of SARS-CoV-2 induced animal models and difficulty in mimicking the complex microenvironments of virus-infected renal cell *in vitro*. Further studies on the protective role of Quercetin and underlying mechanisms are still urgently warranted in order to halt this global pandemic.

As COVID-19 spreads, we are now on the road of discovering specific medicines and vaccines, the safety and efficacy of these promising candidates still wait for verification by clinical trials in the future. The challenge moving forward is to translate these potential preclinical findings into effective therapeutic agents for the treatment of COVID-19 disease and its complications.

## Supporting information

S1 Fig Chemical structure and molecular formula of Quercetin. (DOCX)

S1 Table Pharmacological and molecular properties of Quercetin. (DOCX)

S2 Table Binding energy of Quercetin with the target proteins ACE2 and COVID-19 main protease 3CL. (DOCX)

S1 Dataset (RAR)
