# Supplementary figures and images for: Quercetin as a potential treatment for COVID-19-induced acute kidney injury: Based on network pharmacology and molecular docking study
Source: PLoS One. 2021 Jan 14;16(1):e0245209. doi: 10.1371/journal.pone.0245209 (PMC7808608; doi:10.1371/journal.pone.0245209)

**
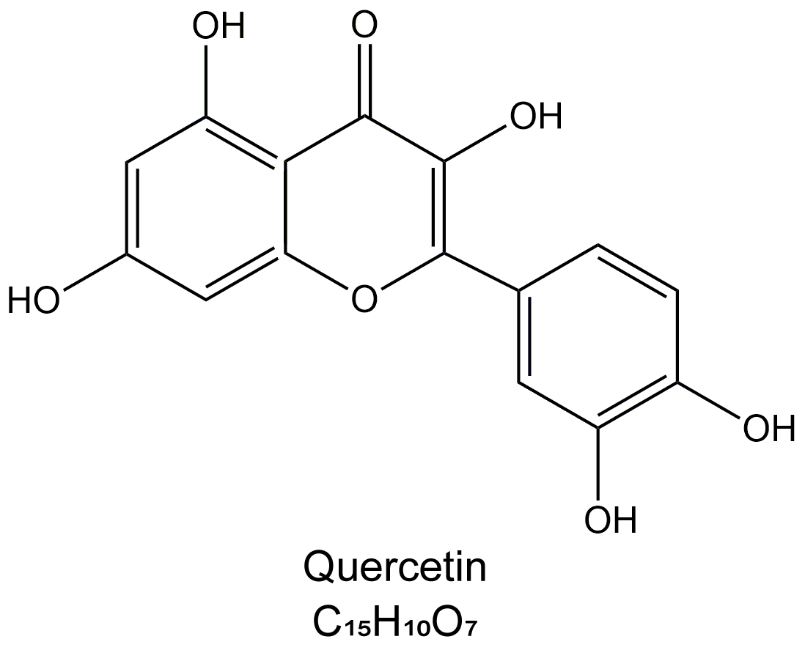
**

**S1 Fig. Chemical structure and molecular formula of Quercetin.**

Supplement: S1 Fig — (DOCX) [file pone.0245209.s001.docx]
