# Supplementary material for: Quercetin as a potential treatment for COVID-19-induced acute kidney injury: Based on network pharmacology and molecular docking study
Source: PLoS One. 2021 Jan 14;16(1):e0245209. doi: 10.1371/journal.pone.0245209 (PMC7808608; doi:10.1371/journal.pone.0245209)
Supplement: S1 Table — (DOCX) [file pone.0245209.s002.docx]

**S1 Table. Pharmacological and molecular properties of Quercetin.**

| **MW** | **AlogP** | **Hdon** | **Hacc** | **OB (%)** | **Caco-2** | **BBB** | **DL** | **FASA** | **TPSA** | **RBN** | **HL** |
| --- | --- | --- | --- | --- | --- | --- | --- | --- | --- | --- | --- |
| 302.25 | 1.5 | 5 | 7 | 46.43 | 0.05 | -0.77 | 0.28 | 0.38 | 131.36 | 1 | 14.4 |

Abbreviations: Caco-2, Caco-2 permeability; OB, oral bioavailability; DL, drug likeness; BBB, blood–brain barrier.
