# Supplementary material for: Quercetin as a potential treatment for COVID-19-induced acute kidney injury: Based on network pharmacology and molecular docking study
Source: PLoS One. 2021 Jan 14;16(1):e0245209. doi: 10.1371/journal.pone.0245209 (PMC7808608; doi:10.1371/journal.pone.0245209)
Supplement: S2 Table — (DOCX) [file pone.0245209.s003.docx]

**S2 Table. Binding energy of Quercetin with the target proteins ACE2 and COVID-19 main protease 3CL.**

| Compound | Binding Energy with ACE2 (1R42) (kcal·mol-1) | Binding Energy with COVID-19 main protease 3CL (6LU7) (kcal·mol-1) |
| --- | --- | --- |
| Quercetin | -3.78 | -4.53 |
